# Weather Woes? Exploring Potential Links between Precipitation and Age-Related Cognitive Decline

**DOI:** 10.3390/ijerph17239011

**Published:** 2020-12-03

**Authors:** Jessica Finlay, Anam Khan, Carina Gronlund, Ketlyne Sol, Joy Jang, Robert Melendez, Suzanne Judd, Philippa Clarke

**Affiliations:** 1 Social Environment and Health Program, Survey Research Center, Institute for Social Research, University of Michigan, 426 Thompson Street, Ann Arbor, MI 48104, USA; anammk@umich.edu (A.K.); gronlund@umich.edu (C.G.); joyjang@umich.edu (J.J.); melendez@umich.edu (R.M.); pjclarke@umich.edu (P.C.); 2 School of Public Health, University of Michigan, 1415 Washington Heights, Ann Arbor, MI 48109, USA; 3 Department of Psychology, University of Michigan, 530 Church Street, Ann Arbor, MI 48109, USA; ksol@umich.edu; 4 School of Public Health, University of Alabama at Birmingham, 1665 University Blvd, Birmingham, AL 35233, USA; sejudd@uab.edu

**Keywords:** cognitive function, longitudinal, aging, environment, climate

## Abstract

Rain, snow, or ice may discourage older adults from leaving their homes with potential consequences for social isolation, decreased physical activity, and cognitive decline. This study is the first to examine potential links between annual precipitation exposure and cognitive function in a large population-based cohort of older Americans. We examined the association between precipitation (percent of days with snow or rain in the past year) and cognitive function in 25,320 individuals aged 45+ from the Reasons for Geographic and Racial Differences in Stroke Study. Linear mixed models assessed the relationship between precipitation and cognitive function, as well as rates of change in cognitive function with age. We found a non-linear relationship between precipitation and cognitive function. Compared to those exposed to infrequent precipitation (less than 20% of days with rain/snow in the past year), cognitive function was higher among older adults experiencing moderately frequent precipitation (20–40% of annual days with precipitation). However, beyond more than about 45% of days with precipitation in the past year, there was a negative association between precipitation and cognitive function, with faster rates of cognitive decline with age. These exploratory findings motivate further research to better understand the complex role of precipitation for late-life cognitive function.

## 1. Introduction

Weather and climate are important determinants of everyday mood, behavior, and health. There is an emerging body of literature seeking to better understand the role of the natural environment on cognitive health and potential environmental risk factors for Alzheimer’s Disease and Related Dementias (ADRD). Population-based studies have found an association between cumulative exposure to air pollution and poor cognitive health [1,2,3], and a recent meta-review for dementia risk factors included air pollution and particulate pollutants [4]. Heat waves, heat stress, and higher indoor temperatures have been linked to worse cognitive functioning [5,6,7]. Studies have found associations between decreased exposure to sunlight and increased probability of cognitive impairment [8,9]. In a study of college students [10], pleasant weather was linked to improved mood and broadened cognition (openness to new information and flexibility in thinking). In recent literature examining the effects of seasonality (changes in seasons throughout the year) on cognition, one study [11] noted robust associations between the season during which a cognitive test was administered and test scores, including diagnosis of mild cognitive impairment, adjusted for a multitude of other physiological and biological confounders. Global cognitive function was higher in the summer and fall compared to winter and spring (with the difference equivalent in cognitive effect to 4.8 years’ difference in age), and the odds of meeting criteria for mild cognitive impairment or dementia were higher in the winter and spring.

There is currently a lack of research examining how exposure to everyday weather conditions may affect cognitive function and trajectories of cognitive decline. It is critical to investigate the climate and weather contexts in which individuals develop and navigate cognitive decline as they age. Older adults in major American cities such as Cleveland, Detroit, Miami, Seattle, Pittsburgh, Portland, and Buffalo experience more than 135 wet days per year [12]. Winters in the Northern Hemisphere are often characterized by prolonged periods of rain and/or snowfall on the ground.

Older adults are particularly vulnerable to climatic conditions given physiological and social factors associated with later life function, such as comorbid health conditions, social isolation, and lack of transportation [13,14]. These vulnerabilities have been characterized with regard to increased extreme heat susceptibility and exposure [15]. Studies from multiple countries have found that rain and snow keep older adults homebound and make otherwise walkable and drivable streets more challenging to navigate. In Vancouver (Canada), precipitation modified walking patterns for older adults, and car-dependent neighborhoods became inaccessible in snow [16]. Heavy rain can make sidewalk cracks and uneven sidewalks indistinguishable, and dirt or grass paths dangerously slippery. Facing ice, older adults have twice the odds of having great difficulty leaving home and curtailing work/volunteer activities, and three times the odds of difficulty driving when compared to younger adults [17]. Over 37 percent of older adults in Toronto (Canada) reduced outdoor walking in slippery conditions [18], and in Detroit (USA), older adults similarly reported fears of falling on ice, challenges of uncleared sidewalks, and the discouragement of rainy weather to walking [19].

Snowy and icy winter conditions in Finland significantly increased odds for loneliness among community-dwelling older people, even after adjusting for restricted mobility, living alone, and health [20]. In five Norwegian communities, older adults indicated lower activity levels (as measured by number of trips taken and kilometers traveled) when compared to summer, resulting in fewer trips to shops, friends, and relatives [21]. Older adults in Minneapolis (USA) voiced concerns and experiences of harm in icy and snowy weather conditions including falls and vulnerability, mobility restrictions, worry and fear, boredom and stress, social exclusion, and isolation [22]. However, participants also noted positive perceptions, such as enjoying distinct seasons and activities (e.g., skiing, hiking), enjoying weather-related “small talk”, bonding with others during inclement weather, and opportunities for socialization and neighborly support.

In summary, there are multiple pathways by which precipitation could be related to cognitive function in older adults, including affecting physical/general activity levels and social engagement. Precipitation can moderate hot temperatures, which may motivate physical activity and boost opportunities for social engagement and support. However, the absence of research on the relationship between precipitation and cognition prevents an understanding of these pathways, and hampers the ability to identify which older adults are at greatest risk for cognitive decline due to differences in exposure to rainy and snowy conditions. This study is the first to examine potential links between annual precipitation and cognitive function using data from the Reasons for Geographic and Racial Differences in Stroke (REGARDS) study. We tested the relationship between annual exposure to precipitation and age-based trajectories of cognitive function in this national cohort of older Americans followed since 2003. We hypothesized that greater exposure to precipitation would be negatively associated with cognitive functioning and with faster rates of cognitive decline with age.

## 2. Methods

### 2.1. Study Sample

The REGARDS study is an ongoing national cohort study examining regional and racial differences in stroke and cognitive function (described in detail by Howard and colleagues [23]). Using mail and telephone contact methods, community-dwelling adults (aged 45+) across the United States were recruited from January 2003 to October 2007. The cohort includes 30,239 non-Hispanic Black and non-Hispanic White individuals with a mean age of 64 years at study enrollment. At baseline, a telephone interview collected information on self-reported socio-demographic, behavioral and lifestyle information, and medical history. An expanded cognitive battery including word list learning/recall and a verbal fluency assessment was introduced in 2006 and conducted during follow-up telephone calls every 2 years thereafter. Study investigators document and verify residential address over the follow-up period and geocode addresses to latitude and longitude coordinates. The University of Alabama in Birmingham annually reviews and approves study procedures (IRB-020925004).

The present study used data from participants who completed at least one cognitive assessment between 2006 and 2017. Individuals without georeferenced address information were excluded. We also excluded participants who reported stroke prior to the first cognitive assessment, due to the known impact of stroke on cognitive function [24]. Our final sample included 25,320 participants contributing a total of 86,715 cognitive assessments across the 12-year follow up period.

### 2.2. Measures

Global Cognitive Function. Measures of verbal learning, memory, and executive function were administered biannually beginning in 2006 using the Consortium to Establish a Registry for Alzheimer’s Disease (CERAD) Word List Learning (WLL) and Word List Delayed Recall (WLD), Animal Fluency Test (AFT) and Letter Fluency Test (LFT) [25,26]. The WLL measures verbal learning (score range, 0–30) and the WLD measures verbal memory (score range, 0–10). The AFT and LFT assess language and executive function: complex cognitive processing used in problem solving or complex action sequences. Scores are based on the number of unique animals (AFT) and unique words beginning with “F” (LFT) named in 1 min. In addition, the 5-min battery recommended by the National Institute of Neurological Disorders and Stroke and Canadian Stroke Network Vascular Cognitive Impairment Harmonization Standards [27] was administered beginning in 2009, consisting of selected subtests of the Montreal Cognitive Assessment ((MoCA), [28]): 5-word delayed memory recall and 6-item orientation (total score range, 0–11).

We did not have a hypothesis for which specific cognitive function domains may be associated with precipitation. Therefore, to capture global cognitive function and use multiple sources of information from the REGARDS cognitive assessment with minimal measurement error, we created a composite index [29,30]. Composites of global cognitive function are robust measures of cognitive function in older adults [31]. We used a factor score derived from a confirmatory factor analysis of all 5 cognitive tests (WLL, WLD, AFT, LFT, MoCA) across all assessments in the REGARDS follow-up period (see Supplementary Materials Figure S1). Factor loadings ranged from 0.43 (MoCA) to 0.79 (AFT), and model fit improved when allowing for correlated error among the memory items (WLL, WLD, MoCA) (Root Mean Square Error of Approximation = 0.013; Comparative Fit Index = 0.999). Standardized factor scores from this model (mean ± standard deviation) were output for each participant at each assessment and used in all subsequent analyses.

Precipitation. We obtained data on daily precipitation for 2006–2017 from the National Oceanic and Atmospheric Administration ((NOAA), [32]). We created person-specific exposures to precipitation in the 365 days preceding each cognitive assessment based on data from the NOAA weather stations located in each participant’s county of residence across the study follow-up period. A summary measure of annual precipitation captured the percentage of days in the past 365 days in which any rain or snow (at least 0.01 mm) was reported by at least 50 percent of the weather stations in the county of a participant’s geocoded residential address. Counties had to provide at least 10 months of valid precipitation data to be included in each annual summary measure.

**Figure 1:**
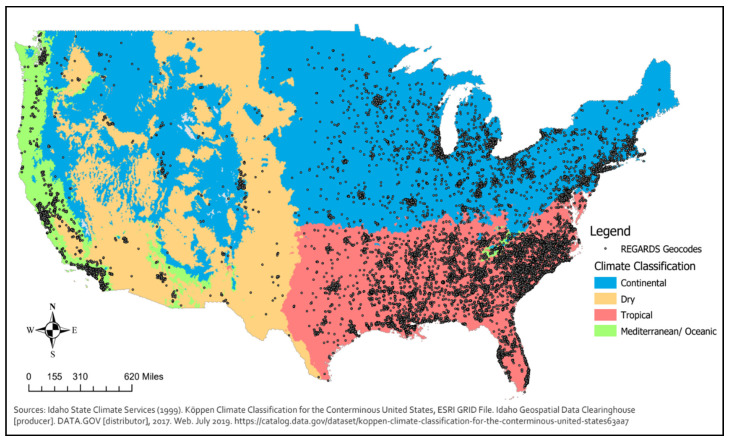
Köppen Climate Region Reclassifications and Reasons for Geographic and Racial Differences in Stroke Study Baseline Geocodes (*N* = 25,320).

Covariates. We adjusted models for individual demographic characteristics (collected at baseline) known to be associated with cognitive function, including gender, race (Black or White), highest level of attained education (less than high school, high school diploma, some college, college degree or higher), and year of entry into the study (baseline year). To account for geographically varying precipitation patterns, we adjusted for climate regions using the Köppen climate classification system, which groups climates based on a region’s seasonal precipitation type and temperature [33]. As illustrated in Figure 1, we further collapsed these types into four broad climate regions (dry, continental, tropical, and Mediterranean/oceanic) based on the geographic distribution of REGARDS study participants at baseline.

### 2.3. Statistical Analysis

We used linear mixed models [34] to examine the relationship between precipitation and trajectories of cognitive function over the 12-year study period. The cognitive function factor score was normally distributed, justifying the linear model. Age was used as the indicator of time (based on age at each cognitive assessment). To facilitate parameter interpretation, we centered time at the youngest age in the data (age 45). We estimated a sequence of two-level models with multiple observations nested within persons over time. Model 1 estimated the unconditional trajectory of cognitive function, including testing and fitting for non-linear rates of change with age. Model 2 added the time-varying measures of annual precipitation. Both intercept and slope effects (interaction between precipitation and age) were tested to assess the relationship between precipitation and levels of cognitive function, as well as on rates of change in cognitive function with age. Model 3 added the socio-demographic and climate region variables to account for factors that could be related to both cognitive function and residence in areas with different weather patterns across the United States.

All models included random effects for both intercepts and slopes to allow cognitive trajectories to vary by person. Nested models were compared according to two goodness-of-fit indices: the Akaike Information Criterion (AIC) and Bayesian Information Criterion (BIC), which make adjustments for model parsimony [35]. Increasingly smaller values indicate good-fitting, parsimonious models. Linear mixed models were estimated using the nlme package in R [36] using full information maximum likelihood. An advantage of this modeling approach was that it allowed for participants with as little as one cognitive assessment to contribute to the model.

Due to the known non-linear relationship between climate and health (e.g., [37]), we modeled the relationship between precipitation and cognition using natural cubic splines. Unlike polynomial terms and piecewise linear splines, natural cubic splines are more appropriate for modeling climate effects because they reduce the number of coefficients and linearly interpolate at the edges of the distribution [38]. These methods more appropriately model differences at the extremes of the distribution where climate effects are often most pronounced. Using the onebasis function in R [39], we estimated models with one to six degrees of freedom (see Figure S2). We used two criteria to determine the optimal number of degrees of freedom in modelling the relationship between precipitation and cognition: (1) the functional form (i.e., the shape of predictive plots), and (2) changes in AIC and BIC. In our data, the functional form of the relationship did not change for models with 3 to 6 degrees of freedom. Further, the BIC decreased from 1 to 3 degrees of freedom, and increased thereafter. We therefore selected the model with two knots and three spline segments (3 degrees of freedom) and included this in the mixed models to capture the non-linear relationship between precipitation and cognitive function.

## 3. Results

Table 1 reports descriptive statistics for the study sample (see Table S1 for additional information). The mean cognitive function factor score for participants was 0.0009 (SD = 2.34). The average age at baseline was 64 years (SD = 8.71, range 45–94 years old). About 39% of the sample was Black and more than half female (56%). Two thirds of the sample had at least some college education while 11% had less than a high school education. On average across the study period, participants were exposed to about a third of annual days with precipitation (mean = 31%, SD = 9.05) with a range of 0–66% of days with precipitation in the past year. Two thirds of the sample (66%) lived in a tropical climate region (located largely in the Southeastern United States) and 24% lived in a continental climate region (most in the Midwest and Northeast). A small proportion (about 2% of the sample) lived in a dry climate region (located largely in the Southwestern states) and 7% lived in Mediterranean/oceanic climate regions (primarily near the Pacific coast).

Table 2 presents the results from the linear mixed models. Model 1 includes the intercept and rate of change in cognitive function with age in the unconditional model. A nonlinear quadratic term for age significantly improved model fit over a strictly linear model (results not shown), indicating that cognitive function initially increased beginning at age 45 but then declined exponentially with increasing age. There was also significant variance in the intercept and rate of change in cognitive function between persons over time (see lower panel of Table 2 for random effects for intercept and slope terms). Model 2 adds the precipitation natural cubic splines. There were significant associations between annual precipitation exposure and both levels of cognitive function and rates of change in cognitive function with age. Because natural cubic splines cannot be interpreted directly [38], Table 2 presents the effects at the 10th and 90th percentiles of precipitation (corresponding to 19% and 41% of days with precipitation in the past year, respectively) compared to the median value (32% of days in the previous year).

There was no significant interaction between precipitation and the quadratic form of age, so models included only the interaction between the precipitation splines and the linear form of age. Again, because natural cubic splines cannot be interpreted directly, the association between precipitation and the linear age slope is presented in Table 2 at both the 10th and 90th percentiles of annual precipitation and at different values of age (60 and 80 years, vs. 70 years) (Table 2). The final model (Model 3) includes the socio-demographic and climate region variables as covariates to account for underlying selection of individuals at greater risk for worse cognitive function into potentially more adverse weather environments. Due to the complexities in directly interpreting these coefficients with natural cubic splines, we illustrate the predicted relationships between cognition, age and precipitation in the figures (based on predicted values from Model 3; Table 2).

**Figure 2:**
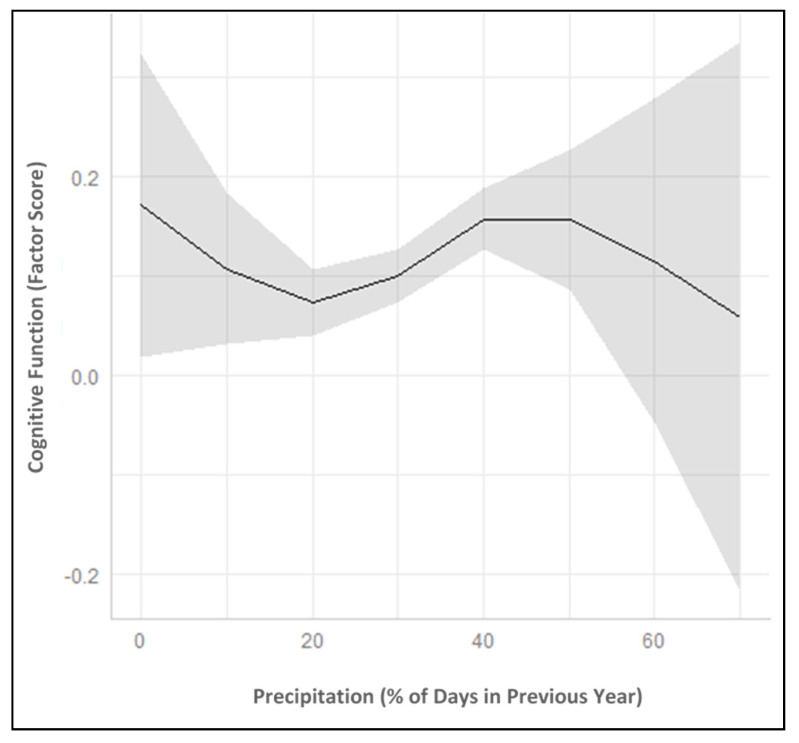
Predicted Association between Precipitation and Cognitive Function: Reasons for Geographic and Racial Differences in Stroke Study (2003–2017). Note: Predicted values are derived from Model 3 (Table 2), plotted at the median value for age, and the rest of covariates at their mean.

Figure 2 presents the predicted values of cognitive function across the range of precipitation experienced in the study population (0–66% of days with rain/snow in the past year), holding age at the median and the rest of covariates at their mean. When precipitation is infrequent (less than 20% of days in the past year), there is an inverse association between precipitation and cognitive function. As the percentage of annual days with precipitation increases from 0% to 20%, cognitive function decreases in these mostly dry conditions. This may be because older adults (and/or their communities) are less equipped to manage rainy/snowy days when it is less common, with potential consequences for social isolation, physical inactivity, and cognitive function. However, there are wide confidence intervals around these predicted values reflecting the small number of individuals experiencing these mostly dry conditions.

Predicted values become more precise when precipitation ranges from 20–40% of days in the past year, which is typical for most of the study population. At this frequency of precipitation there is a positive association with cognitive function. A greater percentage of days with rain/snow over this moderate range of precipitation is associated with higher cognitive function among older adults, net of climate region and demographic characteristics. This is counter to our hypothesis, but may reflect the cognitive benefits of precipitation through more green space (e.g., leafy trees and vegetation to encourage walking outdoors and improve air quality) or the cognitive stimulation afforded by changing seasons and weather.

However, beyond more than about 45% of days with annual precipitation, the association with cognitive function is no longer positive. The relationship is relatively flat across 50–60% of days with precipitation, and exposure to a very high frequency of annual precipitation (between 50–66% of days in the past year) is negatively associated with cognitive function. The relatively wide confidence bands around these predicted values reflect the rarity of these exposures, but for older adults who experience these heavy rainy/snowy conditions, cognitive function is lower with progressively more precipitation exposure.

**Figure 3:**
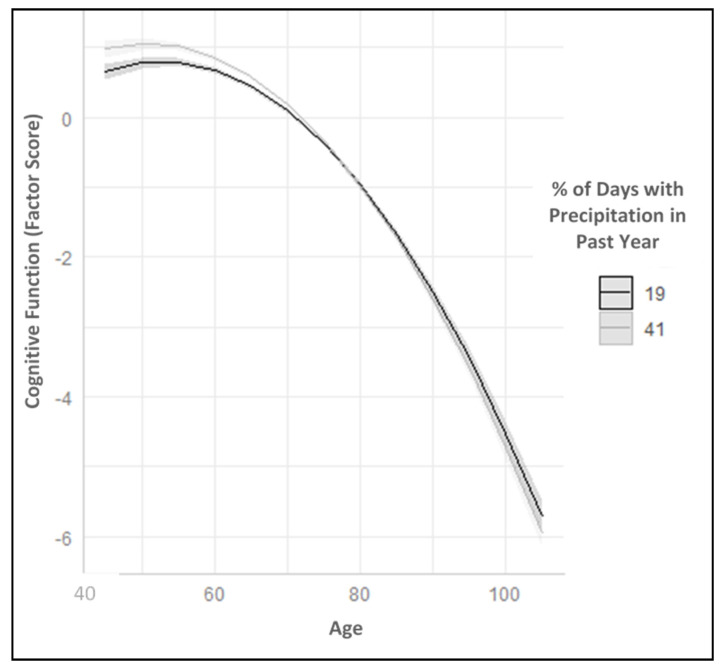
Predicted Trajectory of Cognitive Function over Mid to Late Adulthood by Annual Precipitation Exposure: Reasons for Geographic and Racial Differences in Stroke Study (2003–2017). Note: Predicted trajectories are plotted at the 10th and 90th percentiles of precipitation exposure (corresponding to 19% and 41% of days with precipitation in the past year, respectively).

Figure 3 depicts the predicted relationship between precipitation exposure and rates of change in cognitive function with age (based on coefficients from Model 3, Table 2). Predicted cognition scores over mid to late adulthood are illustrated at exposure to two extremes of precipitation in our data: at the 10th percentile (corresponding to 19% of days with precipitation in the past year) and at the 90th percentile (corresponding to 41% of days with precipitation in the past year). As illustrated in Figure 3, cognitive function is, on average, higher among aging adults who are exposed to moderate annual precipitation than those exposed to less frequent precipitation. However, exposure to these moderate levels of precipitation is associated with a faster rate of decline in cognitive function with age. Beginning at around age 50, adults exposed to more frequent precipitation start to experience more rapid rates of decline in cognitive function that effectively begin to overlap with those experiencing less precipitation by around age 70. Predicted cognitive function continues to decline rapidly for both groups from age 75 onwards.

## 4. Discussion

The current study is the first to explore the association between precipitation and cognitive function using longitudinal survey data on a community-dwelling nationwide cohort of older adults. A growing body of literature looks beyond individual risk factors to consider the role of the social and physical environment for cognitive health [40,41,42,43,44,45,46,47]. However, to date no research to our knowledge has examined the role of natural everyday weather environments for cognitive function. Rain, snow, or ice may discourage older adults from leaving their homes due to the increased walking and driving hazards posed by precipitation (e.g., [17,22]). The social isolation from remaining indoors may in turn reduce cognitive function, given that cognition has been found to decline with social isolation in some large longitudinal studies, due perhaps to increased stress or reduced mental stimulation (e.g., [48]). Physical inactivity and low social contact are both risk factors for dementia [4,49]. By using repeat measures of cognitive function and annual precipitation exposures for a cohort of older adults living across the United States, this study examined differences in associations across time and space.

We found complex relationships where the frequency of exposure to precipitation had both positive and negative consequences for later life cognitive function depending on the magnitude of the exposure. In very dry conditions with infrequent precipitation (less than 20% of days in the past year), we observed a negative association with cognitive function. Increasing precipitation in this dry range may have negative consequences for cognition if rain or snow is unexpected or uncommon, leading to stress, physical inactivity, and isolation among older adults. Community infrastructure may also not be sufficiently equipped to manage rare precipitation events (e.g., inadequate snow removal equipment) resulting in disrupted transportation systems and store closings that may adversely impact older residents. Older adults living in communities with fewer climate-resilient characteristics may be much more vulnerable to precipitation impacts on cognition. For example, in Vancouver (Canada) snow-associated reductions in walking were strong in car-dependent neighborhoods [16]. However, the wide confidence bands around our predicted results at this range of exposure reflect the rarity of such conditions and begs future research to explore these relationships further.

Most older adults in this study experienced, on average, 20–40% of days of annual precipitation. Contrary to our expectations, this was positively associated with cognitive function net of sociodemographic and geographic covariates. Perhaps precipitation in these areas contributes to more lush and green spaces, which can encourage older adults to be more physically active. Rain may help moderate hot temperatures, such as the cooling effect of a thunderstorm on a hot summer day. Precipitation may improve air quality and reduce resident exposure to atmospheric pollution [50,51,52]. Qualitative studies observe older adults enjoying distinct seasons and activities related to precipitation, such as taking walks and playing in puddles with grandchildren [53] and enjoying snowfall around winter holidays. Precipitation can provide older adults with occasions for socialization and support, including weather-related small talk, bonding during harsh winter weather, and receiving assistance from and/or helping neighbors, friends, and family [22]. Precipitation may also motivate physically active seasonal activities, such as skiing and hiking, and boost socialization and social support with opportunities for weather-related conversations, family members checking in during inclement conditions, and neighbors helping with the plowing and salting of driveways and sidewalks [22]. Further, precipitation may provide occasions to decrease stress, such as the enjoyment of reading a book on a rainy day or peaceful views of falling and fresh snow.

However, we also found that exposure to very frequent annual precipitation has potential negative consequences for cognitive function among older adults. At the high extremes of precipitation exposure (50–66% of annual days with rain/snow) cognitive function was negatively associated with precipitation. Exposure to higher rates of annual precipitation was also associated with faster rates of decline in cognitive function with age. There are potential lifestyle pathways by which frequent precipitation could have negative effects on the wellbeing of older adults and their cognitive function. Rainy, icy, and snowy conditions can pose barriers to mobility, reduce physical and general activity levels, and increase social isolation and loneliness [16,20,22]. These effects may impact rates of decline in cognitive functioning over time by exacerbating pre-existing unhealthy behaviors (e.g., physical inactivity, social isolation). Precipitation may also generate new risk factors for cognitive decline including physical inactivity; poor cardiovascular health; lack of mental stimulation, learning, and cognitive activity; social isolation [4,49,54,55]. Further, the effects of frequent precipitation sometimes last beyond the actual day of rain or snow, such as flooded roads and pathways, mud and puddles, slippery and icy walkways, and piled snow. A large snowfall, for example, can cause some older adults to be homebound for days to weeks afterwards [22]. For older adults living in high-precipitation areas, such as the Pacific Northwest and Great Lakes regions, perhaps compounded effects of isolation and inactivity during inclement weather negatively impact rates of cognitive decline. Years with extreme precipitation in a coastal area may also represent years in which hurricanes affected the area. The United States studies, following Hurricanes Katrina (2005), Rita (2005), and Sandy (2012) suggest that hurricanes adversely affect mental health [56,57,58]. In turn, cognitive function may be at risk as a result of diminished mental health, increased social isolation, and stress resulting from hurricanes.

The strengths of this study include a diverse national sample with repeat measures of global cognitive function across later adulthood. Study participants lived across multiple climate regions of the United States, allowing us to examine a broad spectrum of exposures to precipitation among Americans aging in different geographic locations. However, a number of limitations should be noted. First, we do not differentiate between the specific type of precipitation (e.g., rain or snow), nor quantity, which the existing literature (e.g., [16]) suggests may have different effects on the behavior and wellbeing of aging adults. Further research should consider differences by type of precipitation given the strong possibility that snow dissuades participation in social activities among older adults more so than rain [16,17]. Second, the models do not include measures of sunlight so the effects of precipitation may be confounded by light exposure [8]. Third, precipitation recorded in over half of weather stations in a county does not guarantee that it rained or snowed at a participant’s home location. Fourth, we are unable to determine if a participant experienced different weather conditions than their primary residential location, such as participants who live in a warmer location for winter months. Fifth, very few participants were exposed to more than half of days with rain or snow in the past year, so these observations at high frequency precipitation values should be interpreted with caution. However, these individuals are those who are potentially at highest risk, and our modeling strategy was specifically designed to detect these effects at the extremes of precipitation. Sixth, individuals who are particularly limited in their activities by precipitation may also have been more likely to expire or drop out of the study earlier, in which case the results may be biased towards the null. Future research should consider effect modification by mobility and health status and consider inverse-probability weighting or other methods to address selective participant drop-out [59]. Finally, the analyses do not account for neighborhood and county-level characteristics that may interact with effects of precipitation, such as quality of sidewalks, investment in snowplowing and salting, level of walkability, proximity to destinations (e.g., groceries, recreational facilities, healthcare), temperature, and air quality. Further research should examine these community features, as well as time-varying individual mediators and moderators such as relationship status, physical and mental health, and social support networks.

Despite these limitations, the complexity of our findings motivates further research to better understand the role of precipitation for late-life cognitive function, including research on potential mediators such as mobility and social engagement during inclement weather. With climate change, extreme weather events are increasing [60]. Under a more dire climate change scenario, heavy precipitation events are projected to occur 30–40% more often in much of the country [60]. There is, therefore, a growing need to understand how weather-related factors impact cognitive function in aging Americans. Future work can build on our findings to examine varying geographic climates among racially and socioeconomically diverse older adults. Adjusting for additional time-varying variables such as sunlight, temperature, walkability, and road maintenance may yield further insights.

Better understanding of how repeated and long-term exposure to rainy, icy, and snowy conditions may affect the cognitive health of aging individuals can generate novel opportunities to intervene in health and wellbeing. While we cannot change the weather, healthcare providers can take the natural environment into consideration when treating patients to consider those at-risk. They can ask targeted questions about behavior and lifestyle during inclement weather and help develop strategies to overcome potentially-reduced mobility, decreased physical activity levels, and social isolation and loneliness. Older adults may join an indoor mall walking program during the winter, for example, set up ridesharing to appointments and social gatherings with services or friends/family more comfortable driving in inclement weather, take fall prevention classes, and acquire mobility aids to help navigate difficult conditions. Public health officials, community service organizations, and urban planners can take steps to ameliorate the built and social environments in which older adults navigate frequent and prolonged wet and snowy weather, such as programs to check in on homebound and isolated older adults; investment in sidewalk plowing, salting, and maintenance; pedestrian infrastructure improvements to facilitate safer mobility in rainy, snowy, and icy conditions; and indoor winter recreation programs. A better understanding of the mediators and modifiers of this precipitation–cognition association can inform a range of possible mobility, physical activity, and social engagement interventions during inclement weather that could potentially maintain and improve cognitive health as people age.

## Figures and Tables

**Table 1:** Descriptive statistics for study sample (*N* = 25,320): Reasons for Geographic and Racial Differences in Stroke Study (2003–2017).

	(Mean ± SD or %)
Cognitive health factor score (baseline)	0.0009 ± 2.34
Age (years) at baseline assessment	64.38 ± 8.71
Year of Baseline interview	
2003	18.23%
2004	31.25%
2005	22.61%
2006	16.20%
2007	11.71%
Black	39.23%
Female	56.16%
Education	
Less than high school	10.84%
High school	25.58%
Some college	26.90%
College or more	36.63%
Precipitation percent of days in past year	30.99 ± 9.05
Köppen climate region at baseline residence	
Dry	2.21%
Continental	24.32%
Tropical	65.76%
Mediterranean/Oceanic	7.71%

Note: 14 participants missing education were dropped from the subsequent analyses.

**Table 2:** Linear Mixed Effects Regression Coefficients for Cognitive Function over Mid to Late Adulthood: Reasons for Geographic and Racial Differences in Stroke Study (2003–2017).

	Model 1	Model 2	Model 3
	Unconditional Growth Model	+ Precipitation	+ Covariates
Fixed Effects	Beta (SE)	Beta (SE)	Beta (SE)
Intercept	−5.99 (0.40) ***	−5.27 (0.59) ***	−5.39 (0.57) ***
Precipitation (% days in past year) ^¶^			
19% of days in past year		0.04 (0.02) *	−0.04 (0.02) *
41% of days in past year		0.09 (0.01) *	0.05 (0.01) *
Black (ref White)			1.05 (0.02) ***
Female (ref Male)			−0.33 (0.02) ***
Education (ref College or More)			
Less than High School			−2.00 (0.04) ***
High School			−1.33 (0.02) ***
Some College			−0.78 (0.02) ***
Baseline Interview Year (ref 2003)			
2004			0.09 (0.03) **
2005			0.10 (0.03) **
2006			0.18 (0.04) ***
2007			0.17 (0.04) ***
Köppen Climate Regions (ref Dry)			
Continental			0.12 (0.08)
Mediterranean/Oceanic			0.12 (0.07)
Tropical			−0.25 (0.07) ***
Rate of Change			
Age (years)	0.26 (0.01) ***	0.25 (0.01) ***	0.25 (0.01) ***
Age^2^	−0.002 (0.0001) ***	−0.002 (0.0001) ***	−0.002 (0.0001) ***
Age (80 years) × Precipitation (19% days) ^§^		0.07 (0.02) *	0.07 (0.02) *
Age (80 years) × Precipitation (41% days) ^§^		−0.04 (0.01) *	−0.03 (0.01) *
Age (60 years) × Precipitation (19% days) ^§^		−0.07 (0.02) *	−0.07 (0.02) *
Age (60 years) × Precipitation (41% days) ^§^		0.04 (0.01) *	0.03 (0.01) *
Random Effects			
Intercept	2.096	2.072	2.614
Slope (Age)	0.028	0.028	0.045
Slope (Age^2^)	0.001	0.001	0.000
Residual	1.308	1.295	1.296
Goodness of Fit Statistics			
AIC	347,347.4	347,242.5	339,931.3
BIC	347,450.4	347,401.8	340,203.0

Notes: SE = standard error; ref = reference category; AIC = Akaike Information Criteria; BIC = Bayesian Information Criteria; *N* = 25,320 (86,715 observations); * *p* < 0.05, ** *p* < 0.01, *** *p* < 0.001; ^¶^ Coefficients represent the difference in cognitive scores at the 10th and 90th percentiles of precipitation (corresponding to 19% and 41% of annual days with precipitation) compared to the median (reference) of 32% of days in the previous year with precipitation (holding age constant at age 70). ^§^ Coefficients represent the added difference in cognitive scores for the given age vs. 70 years (the added “age effect”), among individuals at the given precipitation level. Note: This is the same as the added difference in cognitive score for the given annual precipitation level vs. 32% annual precipitation (the added “precipitation effect”), among individuals at the given age.

## Supplementary Materials

The following are available online at https://www.mdpi.com/1660-4601/17/23/9011/s1, Table S1: Distribution of first observed cognitive function scores by quartile. Figure S1: Confirmatory factor analysis structure for cognitive function. Figure S2: Function form of the precipitation and cognition relationship with precipitation modeled linearly (A), 2 degrees of freedom (B), 3 degrees of freedom (C), 4 degrees of freedom (D), 5 degrees of freedom (E), 6 degrees of freedom (F), 7 degrees of freedom (G). Precipitation modeled using 3 degrees of freedom was selected because the functional form of the relationship did not change with more degrees of freedom and the BIC model fit statistics was larger, indicating worse model fit.
