# Supplementary material for: Weather Woes? Exploring Potential Links between Precipitation and Age-Related Cognitive Decline
Source: Int J Environ Res Public Health. 2020 Dec 3;17(23):9011. doi: 10.3390/ijerph17239011 (PMC7730226; doi:10.3390/ijerph17239011)
Supplement: Supplementary file 1 [file ijerph-17-09011-s001.pdf]

Supplementary Materials

Supplementary Table S1. Distribution of first observed cognitive function scores by quartile.

Cognitive score quartile	N	25th Pctl	Mean	Median	75th Pctl	90th Pctl	95th Pctl	99th Pctl
Q1	6318	24.93	29.67	30.14	35.62	39.73	43.29	49.04
Q2	6540	24.66	29.60	29.86	35.89	40.82	44.38	49.59
Q3	6188	24.93	29.85	30.41	36.16	40.55	44.11	50.68
Q4	6274	24.38	29.42	29.86	35.89	40.55	44.93	50.96

Notes: N = number of observations. Pctl = Percentile. Quartiles of cognitive scores were created using the first cognitive score for an individual. The percentiles, mean, and median refer to the average cognitive score for each quartile.

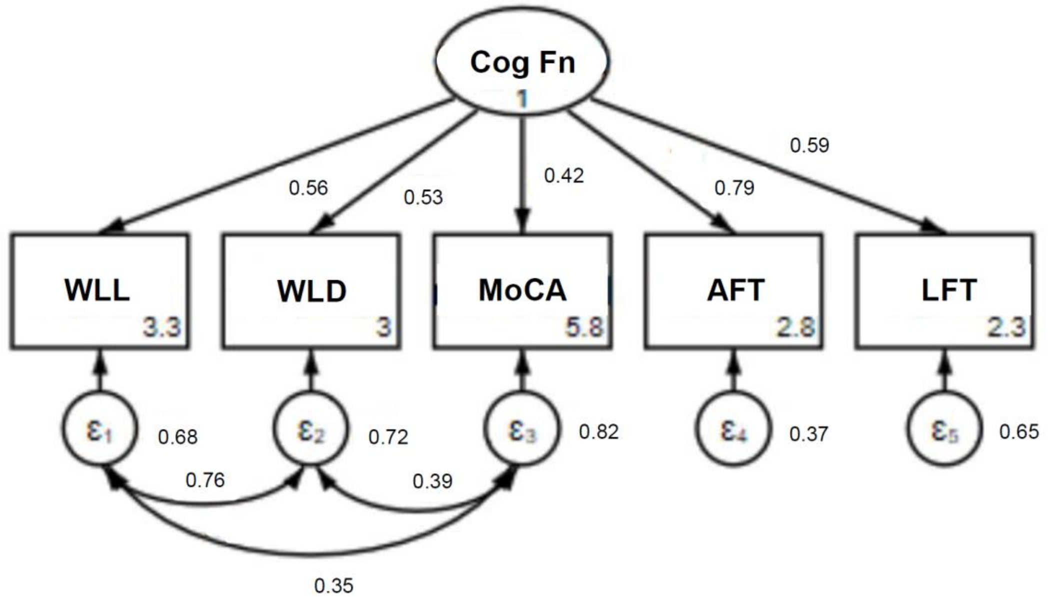

Supplementary Figure S1. Confirmatory factor analysis structure for cognitive function. *Notes:* WLL = Word List Learning, WLD = Word List Delayed, MoCA = Montreal Cognitive Assessment subset, AFT = Animal Fluency Test, LFT = Letter Fluency Test.

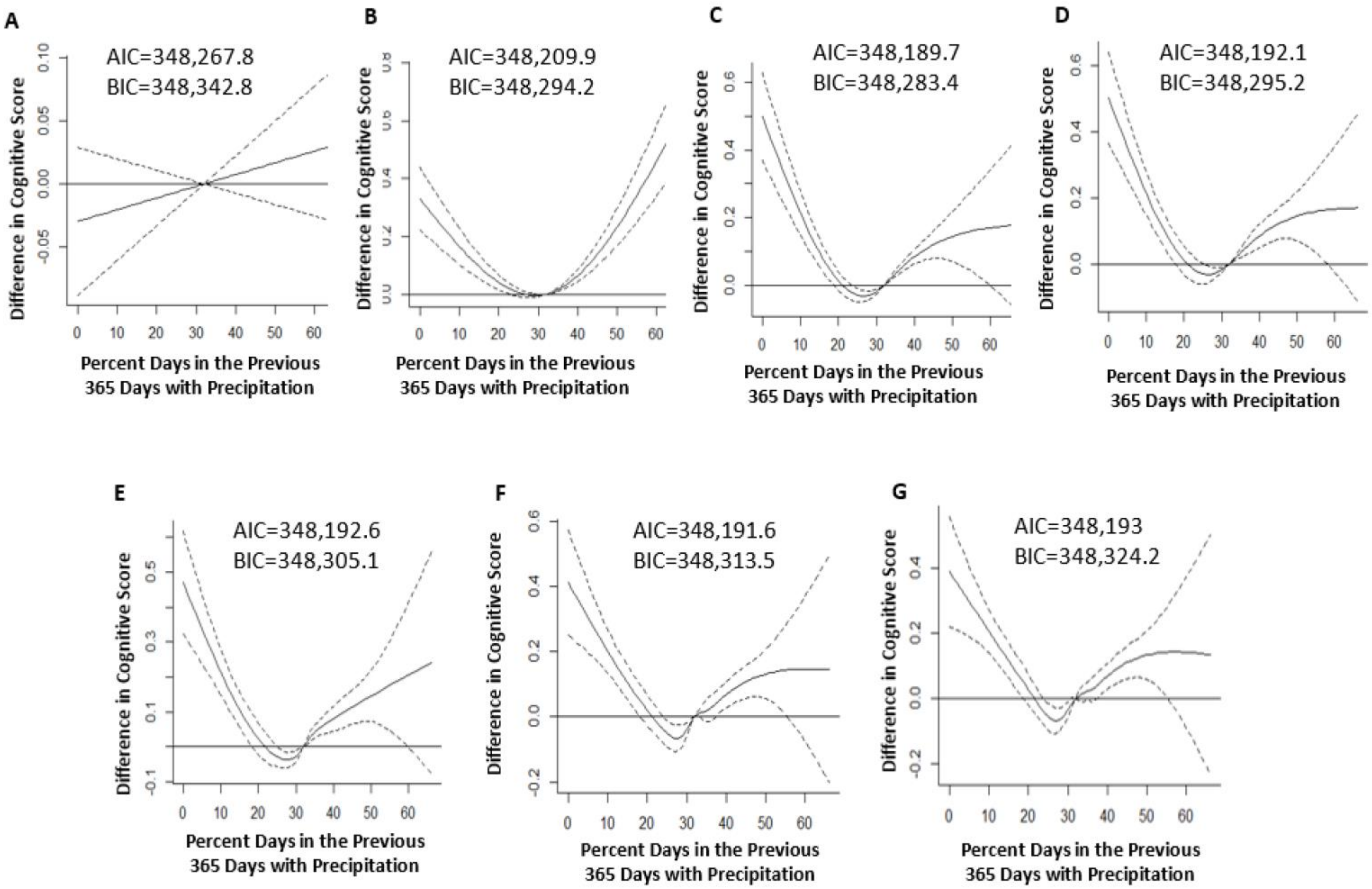

Supplementary Figure S2. Function form of the precipitation and cognition relationship with precipitation modeled linearly (**A**), 2 degrees of freedom (**B**), 3 degrees of freedom (**C**), 4 degrees of freedom (**D**), 5 degrees of freedom (**E**), 6 degrees of freedom (**F**), 7 degrees of freedom (**G**), Precipitation modeled using 3 degrees of freedom was selected because the functional form of the relationship did not change with more degrees of freedom and the BIC model fit statistics was larger, indicating worse model fit.
